# Dexmedetomidine ameliorates memory impairment in sleep-deprived mice

**DOI:** 10.1080/19768354.2019.1688185

**Published:** 2019-11-16

**Authors:** Lakkyong Hwang, Il-Gyu Ko, Jun-Jang Jin, Sang-Hoon Kim, Chang-Ju Kim, Boksoon Chang, Jeong Ho Rho, Eun-Jin Moon, Jae-Woo Yi

**Affiliations:** a Department of Physiology, College of Medicine, Kyung Hee University, Seoul, Republic of Korea; b Department of Pulmonary and Critical Care Medicine, Kyung Hee University Hospital at Gangdong, College of Medicine, Kyung Hee University, Seoul, Republic of Korea; c Department of Medicine, Graduate School, Kyung Hee University, Seoul, Republic of Korea; d Department of Anesthesiology and Pain Medicine, Kyung Hee University Hospital at Gangdong, College of Medicine, Kyung Hee University, Seoul, Republic of Korea

**Keywords:** Sleep deprivation, α_2_-adrenoceptor agonist, dexmedetomidine, inflammation, memory impairment

## Abstract

The selective α2-adrenergic receptor agonist dexmedetomidine acts as an analgesic, sedative, and anesthetic adjuvant. The most common consequence of sleep deprivation is memory impairment. We investigated whether dexmedetomidine can counteract memory impairment caused by sleep deprivation and suppress the production of inflammatory factors. For inducing sleep deprivation, adult male mice were placed inside a water cage containing 15 platforms immersed in water up to 1 cm for 7 days. One day after sleep deprivation, dexmedetomidine at the respective dosage (5, 10, and 20 μg/kg) and α_2_-adrenoceptor antagonist atipamezole (250 μg/kg) were intraperitoneally injected into the mice, once per day for six days. The step-down avoidance task and the Morris water maze test were performed. Western blot analysis was performed to determine the levels of tumor necrosis factor-α (TNF-α), interleukin (IL)-6, brain-derived neurotrophic factor (BDNF), tyrosine kinase B (TrkB), nuclear transcription factor-κB (NF-κB), inhibitor of κBα (IκBα), and ionized calcium binding adapter molecule I (Iba-1) in the hippocampus. Immunohistochemistry was performed for the determination of Ki-67 and glial fibrillary acidic protein (GFAP) expression in the hippocampal dentate gyrus. Dexmedetomidine ameliorated sleep deprivation-induced deterioration of short-term memory and spatial learning ability. Dexmedetomidine inhibited production of inflammatory mediators caused by sleep deprivation. Dexmedetomidine also prevented the decrease in BDNF, TrkB expression, and cell proliferation induced by sleep deprivation. Dexmedetomidine could be used to counteract the neuropathological effects of sleep deprivation.

## Introduction

Sleep deprivation causes anxiety, depressive symptoms, cognitive decline, and various pathological disorders, which affect the normal functioning of a daily routine. The most common consequence of sleep deprivation is memory impairment (Sterniczuk et al. 2013). The other common consequences of sleep loss include sleepiness, fatigue, and poor cognition.

Sleep loss leads to an increase in the circulating level of tumor necrosis factor-α (TNF-α) and interleukin-1β (IL-1β) (Clinton et al. 2011). Sleep loss increases the production and release of sleep regulatory pro-inflammatory molecules (Zielinski and Krueger 2011). Sleep loss can lead to a decline in spatial memory, neuronal cell proliferation and differentiation, and in brain-derived neurotrophic factor (BDNF) levels (Wadhwa et al. 2017).

The selective α2-adrenergic receptor agonist dexmedetomidine has been used as an analgesic, sedative, and an anesthetic adjuvant (Han et al. 2014; Moon et al. 2018). It has also been reported to exert neuroprotective effects against various brain injuries by inhibiting neuronal cell damage, inflammatory response, and neuronal apoptosis (Hwang et al. 2013; Moon et al. 2018). It induces sedation similar to natural sleep, and is relatively a safe drug that does not induce apoptosis under normal conditions (Han et al. 2014; Park et al. 2017).

The aim of the present study was to investigate whether dexmedetomidine counteracts memory impairment and suppresses the production of inflammatory factors caused by sleep deprivation. In this study, the step-down avoidance task for testing short-term memory and the Morris water maze test for testing spatial learning memory were performed on mice. Western blot analysis for determination of levels of TNF-α, interleukin-6 (IL-6), BDNF, tyrosine kinase B (TrkB), nuclear transcription factor-κB (NF-κB), inhibitor of κBα (IκBα), ionized calcium-binding adapter molecule 1 (Iba-1) and immunohistochemical staining for Ki-67, glial fibrillary acidic protein (GFAP) were conducted.

## Materials and methods

### Experimental animals and treatments

Adult male ICR mice (Orient Co., Seongnam, Korea) weighing 30 ± 2 g (age-15 weeks) were purchased for this research. Animal experimental procedures were approved by the Institutional Animal Care and Use Committee of Kyung Hee University (KHUASP [SE]-16-021).

In the first experiment, to determine the efficacy of dexmedetomidine at various concentrations, the mice were divided into the following five groups (*n* = 8 in each group): control group, sleep deprivation group, sleep deprivation and 5 μg/kg dexmedetomidine-treated group, sleep deprivation and 10 μg/kg dexmedetomidine-treated group, and sleep deprivation and 20 μg/kg-dexmedetomidine treated group.

In the second experiment, after the optimal concentration was selected, a selective α2-adrenergic receptor antagonist was used to evaluate the effect of dexmedetomidine. Mice were divided into the following five groups (*n* = 8 in each group): control group, sleep deprivation group, sleep deprivation and 250 μg/kg atipamezole-treated group, sleep deprivation and 20 μg/kg dexmedetomidine-treated group, and sleep deprivation and 250 μg/kg atipamezole- and 20 μg/kg dexmedetomidine-treated group.

**Figure 1.**
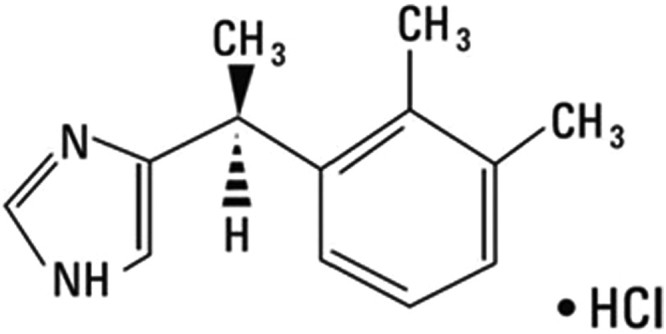
Structural formula of dexmedetomidine.

After one day of sleep deprivation, dexmedetomidine (Precedex, Pfizer, NY, USA) and α_2-_adrenoceptor antagonist atipamezole (Antisedan, Orion Pharma, Espoo, Finland) were injected intraperitoneally into the mice, once a day for six days. Previous studies were considered for determining the required dosages of dexmedetomidine (Hwang et al. 2013; Choi et al. 2017; Moon et al. 2018). Structural formula of dexmedetomidine was shown in Figure 1.

### Induction of sleep deprivation

**Figure 2.**
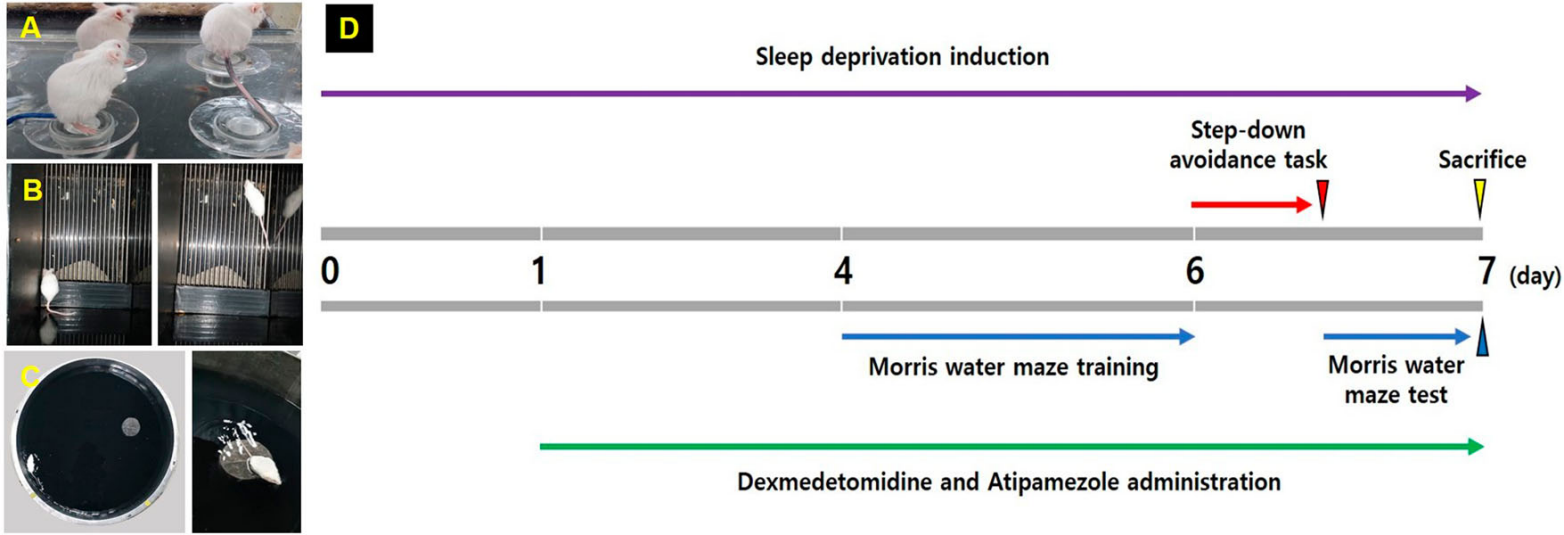
Experimental schedule. (A) Induction of sleep deprivation, (B) step-down avoidance task, (C) Morris water maze test, (D) experimental time line.

Sleep deprivation was induced by placing the animals on a customized platform in an acryl cage for seven consecutive days (Moon et al. 2018). Time schedule of experiment is presented in Figure 2. The acryl cage (90 cm width × 60 cm length × 40 cm height) consisted of 15 platforms (3 cm diameter and 15 cm height). The platform surfaces were immersed in water up to 1 cm and the mice were able to move from one platform to another. Food and water were provided on the grid placed on top of the acryl cage during sleep deprivation.

### Step-down avoidance task

The step-down avoidance task was performed to evaluate short-term memory according to a previously described method (Lee et al. 2018). Six days after sleep deprivation, the mice were trained in a step-down avoidance task. A mouse was placed on a 7 × 25 cm platform that was 2.5 cm in height. The platform faced a grid (45 × 25 cm) of parallel steel bars, 0.1 cm in caliber and spaced 1 cm apart. In the training session, the animal was immediately shocked by a 0.3 mA scrambling foot for 2 sec as it stepped down the grid. After 4 h of training, latency in each group was examined. In the test session, the mouse was again placed on the platform. The time, as determined by the test, elapsed until the mouse stepped down and placed all the four paws on the grid was defined as latency. The maximum latency was counted as 180 sec.

### Morris water maze test

To evaluate spatial learning memory in mice, the Morris water maze test was performed according to a previously described method (Lee et al. 2018). The water maze apparatus was composed of a circular pool (170 cm diameter, 50 cm height) filled with opaque water (black water). Water was filled up to a height of 37 cm and was maintained at a temperature of 22°C. In the water maze, a platform (15 cm diameter, 35 cm height) was located 2 cm below the water surface in one of the four regions in the pool. Clearly visible cues outside the maze were provided for orientation. Training sessions were conducted for three days from the third day of sleep deprivation and were conducted from 1 pm to 5 pm to evaluate the spatial learning memory. In the acquisition phase, the mouse practiced in each zone once a day for three consecutive days to find the platform submerged 2 cm below the water surface. For each trial, the mouse was placed in water, facing the wall of the tank, in one of the four start locations. The mouse was allowed to search for the platform for 60 s. If the mouse found the platform, it was allowed to stay on the platform for 10 s. If the mouse did not find the platform within 60 s, the mouse was guided and allowed to stay on the platform for 10 s. Seven days after sleep deprivation, to assess spatial learning memory, the animals were subjected to the 60 s probe trial, and then the platform was removed from the pool. The mouse was placed in water at the diagonal position on the platform, and the time required to reach the platform was measured. The timings of occupancy on the quadrant platform, swimming speed, and swimming distance were recorded automatically by a video tracking system (SMART; Pan-Lab, Barcelona, Spain).

### Tissue preparation

According to a previously described method (Ko et al. 2018), the mice were sacrificed immediately after the completion of the Morris water maze test. The mice were completely anesthetized by Zoletil 50® (10 mg/kg, intraperitoneally; Vibac Laboratories, Carros, France). Subsequently, 50 mM phosphate-buffered saline (PBS; Duksan General Science, Seoul, Korea) was transcardially perfused, and the mice were fixed using 4% paraformaldehyde in 100 mM phosphate buffer (pH 7.4; Duksan General Science). After dissecting the brains, 40 μm thick coronal sections were fabricated using a freezing microtome (Leica, Nussloch, Germany). On an average, ten sections were selected from the Bregma −1.82 to −2.30 mm in size from each mouse.

### Immunohistochemistry

Immunohistochemistry was performed to visualize the Ki-67 and GFAP expression, as previously described (Park et al. 2017; Lee et al. 2018). After washing the sections using 0.05 M PBS (Duksan General Science), the sections were treated with a 3% H_2_O_2_ (Sigma-Aldrich Inc., St. Louis, MO, USA) and 20% methanol (Duksan General Science). The sections were subsequently treated with anti-mouse Ki-67 antibody (1:500; Novocastra Laboratories, Newcastle, UK) and anti-rabbit GFAP antibody (1:500; Santa Cruz Biotechnology, CA, USA) at 4°C for 48 h, after which they were incubated with biotinylated mouse secondary antibody (1:200; Vector Laboratories, Burlingame, CA, USA) for 2 h at room temperature. The sections were treated with ABC Elite kit (Vector Laboratories) for 2 h followed by treatment with 0.02% diaminobenzidine (Sigma-Aldrich Inc.) and 0.03% H_2_O_2_ (Sigma-Aldrich Inc.) for 5 min. Finally, the coverslips were mounted using Permount® (Thermo Fisher Scientific, New Jersey, NJ, USA).

### Western blot analysis

Protein lysates were extracted according to a previously described method (Hwang et al. 2018; Kim et al. 2018). Dissected hippocampal tissues were homogenized in 400 μl per 1 g concentration of RIPA buffer (Cell Signaling Technology, Beverly, MA, USA) with 1 mM PMSF (Sigma-Aldrich Inc.) on ice. The homogenized sample was incubated on ice for 20 min. Incubated sample was centrifuged at 14,000 g for 10 min at 4°C, and supernatants were collected. Protein content was measured using a micro-drop plate reader (Thermo Fisher Scientific). NF-кB and IкBα in the hippocampus were detected using nuclear/cytosol fractionation kit (BioVision Inc, Milpitas, CA, USA) according to the manufacturer’s instructions. The following primary antibodies (1:1000 dilution) were selected to react overnight at 4°C: mouse anti-β-actin, anti-TNF-α, anti-interleukin-6 (IL-6), anti-proBDNF, anti-BDNF, anti-Iba-1, rabbit anti-TrkB, anti-NF-κB, and anti-IκBα (Santa Cruz Biotechnology). Subsequently, membranes were incubated for 1 h with attempt secondary antibodies (1:2000; Vector Laboratories). Blot membrane was detected using the HRP-conjugated IgG (Vector Laboratories) and the enhanced chemiluminescence detection kit (Bio-Rad, Hercules, CA, USA). Detected bands were quantified by Image-Pro® plus image analysis system (ver. 6.0, Media Cybernetics Inc., Silver Spring, MD, USA).

### Data analysis

The number of Ki-67-positive cells in each area of the hippocampal dentate gyrus was counted according to a stereological method (Kempermann et al. 1997; Kim et al. 2007). The number of Ki-67-positive cells in the hippocampal dentate gyrus was counted according to a stereological method hemilaterally under a light microscope (Olympus, Tokyo, Japan). The total number of Ki-67-positive cells in the granular layer, *N*, was calculated by multiplying the numerical density of Ki-67-positive cells, *N*_v_, with the reference volume (mm^3^), *V*_ref_, as *N* = *N*_v_ × *V*_ref_. *V*_ref_ was estimated according to the Cavalieri's method (Kempermann et al. 1997; Kim et al. 2007) as *V*_ref_ = *a* × *t* × *s*, where *a* represents the mean area of the granular cell layer, *t* the mean thickness of the microtome section (40 μm), and *s* is the total number of sections in the reference volume.

The optical densities of GFAP immunoreactive fibers were measured on 100 × 100 μm^2^ images in the hippocampal dentate gyrus using an image analyzer (Multiscan, Fullerton, CA, USA). The GFAP-positive fiber densities were calculated as follows: optical density of the lesion side/optical density of the intact side.

Statistical analysis was performed using one-way analysis of variance and Duncan’s post-hoc test using SPSS software (ver. 23, IBM Co., Armonk, NY, USA), and the values were expressed as mean ± standard error (SEM). *P* value < 0.05 was considered to indicate a statistically significant difference.

## Results

### Effect of dexmedetomidine on short-term memory

**Figure 3.**
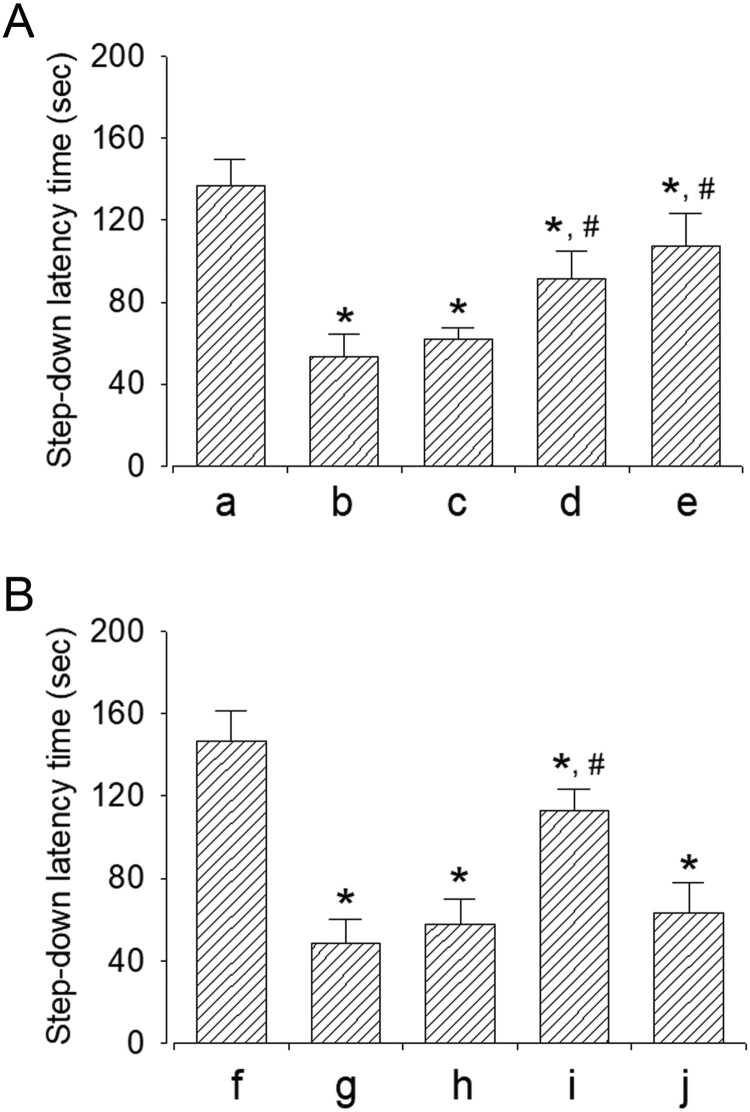
Effect of dexmedetomidine on short-term memory. A: Evaluation of dose-dependent effects of dexmedetomidine on short-term memory (*n* = 8). (a) Control group, (b) sleep deprivation group, (c) sleep deprivation and 5 μg/kg dexmedetomidine-treated group, (d) sleep deprivation and 10 μg/kg dexmedetomidine-treated group, (e) sleep deprivation and 20 μg/kg dexmedetomidine-treated group. B: Evaluation of effect of dexmedetomedine antagonist on short-term memory (*n* = 8). (f) Control group, (g) sleep deprivation group, (h) sleep deprivation 250 μg/kg atipamezole-treated group, (i) sleep deprivation and 20 μg/kg dexmedetomidine-treated group, (j) sleep deprivation and 250 μg/kg atipamezole-treated with 20 μg/kg dexmedetomidine-treated group. * represents *P* < 0.05 compared to the control group. # represents *P* < 0.05 compared to sleep deprivation group.

The efficacy of dexmedetomidine concentration in the step-down avoidance task is shown in Figure 3A. Sleep-deprived mice showed a shorter latency period compared to mice in the control group (*P* < 0.05). However, dexmedetomidine treatment increased the latency period in a dose-dependent manner (*P* < 0.05). The mice in 20 μg/kg dexmedetomidine-treated group showed a significant increase in latency period compared to all other groups (*P* < 0.05).

The results of the step-down avoidance task in mice treated with α2-adrenoceptor antagonist are shown in Figure 3B. Sleep deprivation significantly disturbed short-term memory (*P* < 0.05), whereas dexmedetomidine treatment alleviated sleep deprivation-induced short-term memory impairment (*P* < 0.05). On treatment with dexmedetomidine antagonist atipamezole, the dexmedetomidine-induced improvement in short-term memory was reversed (*P* < 0.05).

### Effect of dexmedetomidine on spatial learning memory

**Figure 4.**
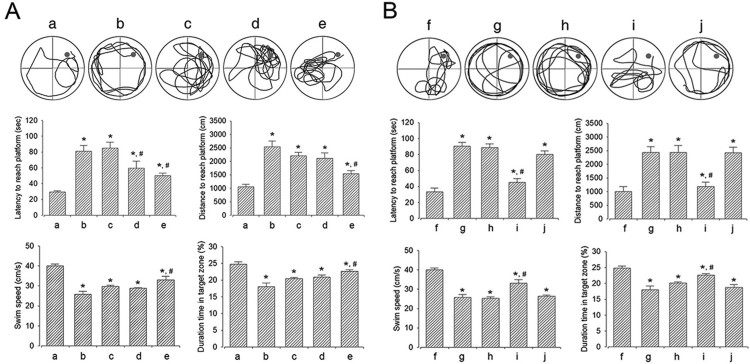
Effect of dexmedetomidine on the spatial learning ability. A: Evaluation of dose-dependent effects of dexmedetomidine on spatial learning ability (*n* = 8). (a) Control group, (b) Sleep deprivation (SD)-induced group, (c) SD-induced and 5 μg/kg dexmedetomidine-treated group, (d) SD-induced and 10 μg/kg dexmedetomidine-treated group, (e) SD-induced and 20 μg/kg dexmedetomidine-treated group. B: Evaluation of effect of dexmedetomidine antagonist on spatial learning ability (*n* = 8). (f) Control group, (g) sleep deprivation group, (h) sleep deprivation 250 μg/kg atipamezole-treated group, (i) sleep deprivation and 20 μg/kg dexmedetomidine-treated group, (j) sleep deprivation and 250 μg/kg atipamezole-treated with 20 μg/kg dexmedetomidine-treated group. * represents *P* < 0.05 compared to the control group. # represents *P* < 0.05 compared to sleep deprivation group.

The efficacy of dexmedetomidine concentration assessed by the Morris water maze test is shown in Figure 4A. Sleep-deprived mice showed a longer latency period and distance, slow swimming speed, and shorter duration of occupancy in the target zone compared to the mice in the control group (*P* < 0.05). However, dexmedetomidine treatment shortened latency period and distance and led to an increased swimming speed and longer duration of occupancy in the target zone in a dose-dependent manner (*P* < 0.05). The mice in the 20 μg/kg dexmedetomidine-treated group showed a significantly shortened latency period and distance and led to an increased swimming speed and longer duration of occupancy in the target zone compared to that in all other groups (*P* < 0.05).

The results of the Morris water maze test in mice treated with α2-adrenoceptor antagonist are shown in Figure 4B. Sleep deprivation significantly disturbed spatial learning memory (*P* < 0.05), whereas dexmedetomidine treatment alleviated sleep deprivation-induced spatial learning memory impairment (*P* < 0.05). On treatment with atipamezole, the dexmedetomidine-induced improvement in spatial learning memory was reversed (*P* < 0.05).

### Effect of dexmedetomidine on inflammatory mediators in hippocampus

**Figure 5.**
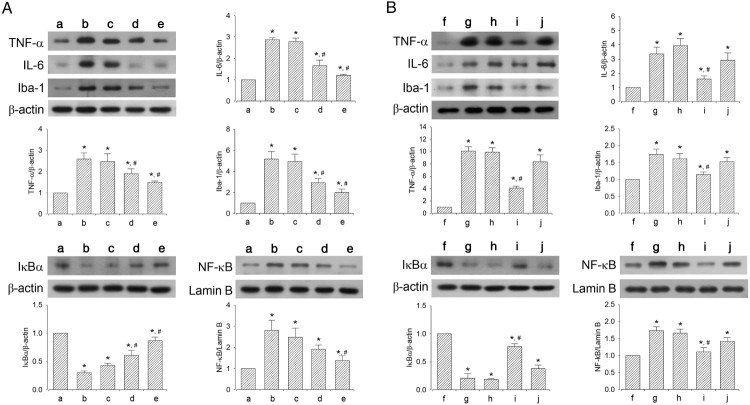
Effect of dexmedetomidine on inflammatory mediators and Iba-1 expression in the hippocampus. A: Evaluation of dose-dependent effects of dexmedetomidine on inflammatory mediators (*n* = 8). (a) Control group, (b) sleep deprivation group, (c) sleep deprivation and 5 μg/kg dexmedetomidine-treated group, (d) sleep deprivation and 10 μg/kg dexmedetomidine-treated group, (e) sleep deprivation and 20 μg/kg dexmedetomidine-treated group. B: Evaluation of effect of dexmedetomidine antagonist on inflammatory mediators (*n* = 8). (f) Control group, (g) sleep deprivation group, (h) sleep deprivation 250 μg/kg atipamezole-treated group, (i) sleep deprivation and 20 μg/kg dexmedetomidine-treated group, (j) sleep deprivation and 250 μg/kg atipamezole-treated with 20 μg/kg dexmedetomidine-treated group. * represents *P* < 0.05 compared to the control group. # represents *P* < 0.05 compared to sleep deprivation group.

The efficacy of dexmedetomidine concentration on inflammatory mediators is shown in Figure 5A. Sleep-deprived mice showed increased levels of TNF-α, IL-6, Iba-1, and NF-κB and decreased levels of IκBα compared to mice in the control group (*P* < 0.05). However, dexmedetomidine treatment suppressed the levels of TNF-α, IL-6, Iba-1, and NF-κB (*P* < 0.05) and increased the level of IκBα in a dose-dependent manner (*P* < 0.05). The mice in the 20 μg/kg dexmedetomidine-treated group showed significantly altered levels of TNF-α, IL-6, Iba-1, NF-κB, and IκBα compared to that in all other groups (*P* < 0.05).

The effects of α2-adrenoceptor antagonist on inflammatory mediators are shown in Figure 5B. Sleep deprivation increased TNF-α, IL-6, Iba-1, and NF-κB expression (*P* < 0.05) and decreased IκBα expression (*P *< 0.05). However, dexmedetomidine treatment reduced TNF-α, IL-6, Iba-1, and NF-κB expression (*P* < 0.05) and increased IκBα expression (*P* < 0.05). On treatment with atipamezole, the decreased levels of TNF-α, IL-6, Iba-1, and NF-κB and increased levels of IκBα caused by dexmedetomidine were reversed (*P* < 0.05).

### Effect of dexmedetomidine on BDNF and TrkB expression in the hippocampus

**Figure 6.**
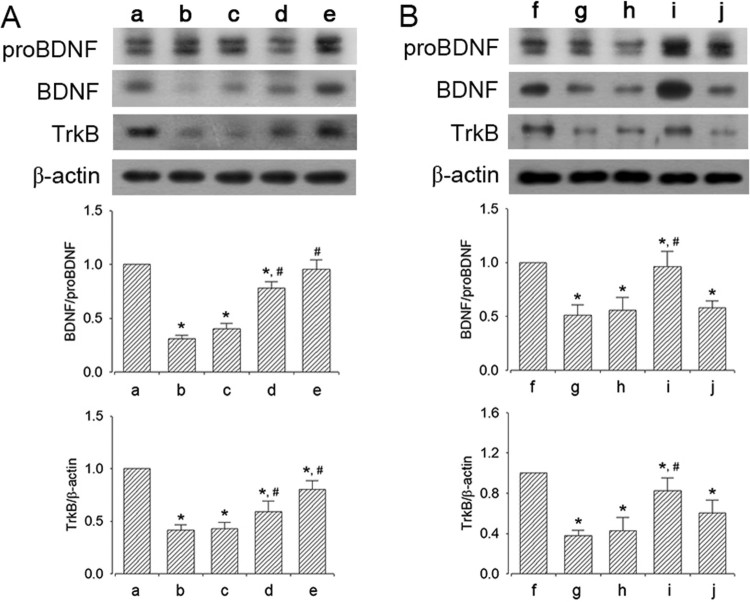
Effect of dexmedetomidine on BDNF and TrkB expression in the hippocampus. A: Evaluation of dose-dependent effects of dexmedetomidine on BDNF and TrkB expression (*n* = 8). (a) Control group, (b) sleep deprivation group, (c) sleep deprivation and 5 μg/kg dexmedetomidine-treated group, (d) sleep deprivation and 10 μg/kg dexmedetomidine-treated group, (e) sleep deprivation and 20 μg/kg dexmedetomidine-treated group. B: Evaluation of effect of dexmedetomidine antagonist on BDNF and TrkB expression (*n* = 8). (f) Control group, (g) sleep deprivation group, (h) sleep deprivation 250 μg/kg atipamezole-treated group, (i) sleep deprivation and 20 μg/kg dexmedetomidine-treated group, (j) sleep deprivation and 250 μg/kg atipamezole-treated with 20 μg/kg dexmedetomidine-treated group. * represents *P* < 0.05 compared to the control group. # represents *P* < 0.05 compared to sleep deprivation group.

The efficacy of dexmedetomidine concentration on BDNF and TrkB expression is shown in Figure 6A. Sleep-deprived mice showed decreased expression of BDNF and TrkB compared to mice in the control group (*P* < 0.05). However, dexmedetomidine treatment enhanced the expression of BDNF and TrkB in a dose-dependent manner (*P* < 0.05). The mice in the 20 μg/kg dexmedetomidine-treated group showed significantly enhanced BDNF and TrkB expression compared to that in all other groups (*P* < 0.05).

The effects of α2-adrenoceptor antagonist on expression of BDNF and TrkB are shown in Figure 6B. Sleep deprivation decreased the expression of BDNF and TrkB (*P* < 0.05), whereas dexmedetomidine treatment enhanced the expression of BDNF and TrkB (*P* < 0.05). On treatment with atipamezole, the increased BDNF and TrkB expression caused by dexmedetomidine was reversed (*P* < 0.05).

### Effect of dexmedetomidine on Ki-67 and GFAP expressions in the hippocampal dentate gyrus

**Figure 7.**
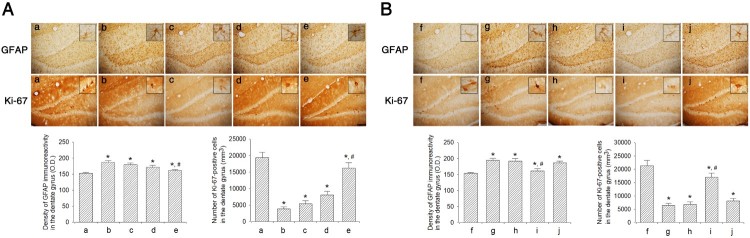
Effect of dexmedetomidine on Ki-67 and GFAP expression in the hippocampal dentate gyrus. A: Evaluation of dose-dependent effects of dexmedetomidine on GFAP and Ki-67 expression (*n* = 8). (a) Control group, (b) sleep deprivation group, (c) sleep deprivation and 5 μg/kg dexmedetomidine-treated group, (d) sleep deprivation and 10 μg/kg dexmedetomidine-treated group, (e) sleep deprivation and 20 μg/kg dexmedetomidine-treated group. B: Evaluation of effect of dexmedetomidine antagonist on GFAP expression and Ki-67 (*n* = 8). (f) Control group, (g) sleep deprivation group, (h) sleep deprivation 250 μg/kg atipamezole-treated group, (i) sleep deprivation and 20 μg/kg dexmedetomidine-treated group, (j) sleep deprivation and 250 μg/kg atipamezole-treated with 20 μg/kg dexmedetomidine-treated group. Insets show Ki-67 and GFAP-positive expression. The scale bars represent 150 μm. Insets are higher magnification (scale bar: 50 μm). * represents *P* < 0.05 compared to the control group. # represents *P* < 0.05 compared to sleep deprivation group.

The efficacy of dexmedetomidine concentration on Ki-67 and GFAP expression in the hippocampal dentate gyrus is shown in Figure 7A. Sleep-deprived mice showed decreased Ki-67 expression and increased GFAP expression compared to mice in the control group (*P *< 0.05). However, dexmedetomidine treatment increased Ki-67 expression and decreased GFAP expression in a dose-dependent manner (*P *< 0.05). The mice in 20 μg/kg dexmedetomidine-treated group showed significantly increased expression of Ki-67 and decreased expression of GFAP compared to that in all other groups (*P* < 0.05).

The effects of α2-adrenoceptor antagonist on expression of Ki-67 and GFAP are shown in Figure 7B. Sleep deprivation decreased Ki-67 expression and increased GFAP expression (*P* < 0.05), whereas dexmedetomidine treatment enhanced Ki-67 expression and decreased GFAP expression (*P* < 0.05). On treatment with atipamezole, the increased Ki-67 and decreased GFAP expression caused by dexmedetomidine were reversed (*P* < 0.05).

## Discussion

Dexmedetomidine provides sedation without increasing the risk of respiratory depression unlike other commonly used sedatives, such as propofol, fentanyl, and midazolam. Dexmedetomidine decreases the activity of noradrenergic neurons in the locus ceruleus of the brain stem, inducing sedation, thereby increasing the activity of gamma-aminobutyric acid neurons in the pre-optic nerve nucleus of the brain (Nelson et al. 2003). In contrast, other sedatives such as propofol and benzodiazepines directly increase the activity of gamma-amino butyric acid neurons (Panzer et al. 2009). Sedation caused by dexmedetomidine is similar to that caused by natural sleep. Therefore, dexmedetomidine shows limited loss of memory than benzodiazepines (Panzer et al. 2009).

Sleep deprivation can cause a decline in spatial memory, in neuronal cell proliferation and differentiation, and in BDNF level accompanied by an upregulation of neuroinflammatory molecules (Wadhwa et al. 2017). Sleep deprivation can induce impairment of spatial learning ability and memory function (Li et al. 2019). In this study, dexmedetomidine ameliorated sleep deprivation-induced deterioration of short-term memory and spatial learning memory.

Sleep-deprived mice exhibited increased IL-1β and TNF-α pro-inflammatory gene expression in brain as well as in peripheral tissues (Ashley et al. 2016). The level of Iba-1 was increased in both the cerebral cortex and the hippocampus in the lipopolysaccharide-injected group compared to that in control group (Hu et al. 2011). Enhanced Iba-1 expression represents microglial activation in sleep deprivation (Wadhwa et al. 2018). NF-κB is present in the neurons and glial cells, and its activation leads to the transcription of many inflammatory molecules such as IL-1β and TNF-α (Faraut et al. 2012). IκBα is inversely related to NF-κB. IκBα plays an important role in regulating NF-κB activity in the brain and a robust NF-κB/IκBα-mediated neuroinflammatory response (Lian et al. 2012). Dexmedetomidine inhibits the IL-1β-induced IL-6 synthesis mediated by the α2-adrenergic receptors (Tanabe et al. 2014). Dexmedetomidine exerts a neuroprotective effect through NF-κB in lipopolysaccharide-induced cognitive dysfunction (Zhang et al. 2018). Dexmedetomidine can suppress hippocampal inflammation caused by surgical trauma and can effectively improve cognitive function after surgery in rats. (Chen et al. 2019). In this study, dexmedetomidine inhibited sleep deprivation-induced production of inflammatory mediators.

Stressful situations such as sleep deprivation suppress BDNF and TrkB expression in the hippocampus, resulting in inhibition of neurogenesis (Sompol et al. 2011). BDNF expression was decreased by sleep deprivation (Sahu et al. 2013). The upregulation of BDNF and TrkB expression is associated with increased cell proliferation in the hippocampus (Jin et al. 2017). In this study, dexmedetomidine prevented the decrease in level of BDNF and TrkB caused by sleep deprivation.

Ki-67 is a marker for cell proliferation in the hippocampus (Kee et al. 2002). New cell formation in the dentate gyrus was decreased by sleep deprivation (Sahu et al. 2013). Prolonged sleep deprivation decreases hippocampal cell proliferation and neurogenesis (Murata et al. 2018). In this study, dexmedetomidine increased cell proliferation inhibited by sleep deprivation.

Dexmedetomidine exerts neuroprotective effect against intracerebral hemorrhage (Hwang et al. 2013) and cerebral ischemia (Choi et al. 2017). Treatment with dexmedetomidine suppresses the expression of apoptosis-related factors released due to brain insults, resulting in improvements in short-term memory and spatial learning memory (Hwang et al. 2013; Choi et al. 2017).

The results of present study showed that dexmedetomidine inhibited production of sleep deprivation-induced inflammatory mediators in the hippocampus. Dexmedetomidine also increases new cell formation in the hippocampus through enhancing BDNF production in sleep deprivation mice. These effects of dexmedetomidine ameliorated sleep deprivation-induced impairment of short-term memory and spatial learning memory. In particular, the efficacy was excellent at high concentration. Based on the present results, dexmedetomidine can be used to counter the neuropathological effects of sleep deprivation.

## References

[CIT0001] Ashley NT, Sams DW, Brown AC, Dumaine JE. 2016 Novel environment influences the effect of paradoxical sleep deprivation upon brain and peripheral cytokine gene expression. Neurosci Lett. 615:55–59. doi: 10.1016/j.neulet.2016.01.013 26806035 PMC4755797

[CIT0002] Chen N, Chen X, Xie J, Wu C, Qian J. 2019 Dexmedetomidine protects aged rats from postoperative cognitive dysfunction by alleviating hippocampal inflammation. Mol Med Rep. 20:2119–2126. 31257507 10.3892/mmr.2019.10438 PMC6691222

[CIT0003] Choi IY, Hwang L, Jin JJ, Ko IG, Kim SE, Shin MS, Shin KM, Kim CJ, Park SW, Han JH, et al. 2017 Dexmedetomidine alleviates cerebral ischemia-induced short-term memory impairment by inhibiting the expression of apoptosis-related molecules in the hippocampus of gerbils. Exp Ther Med. 13:107–116. doi: 10.3892/etm.2016.3956 28123477 PMC5244889

[CIT0004] Clinton JM, Davis CJ, Zielinski MR, Jewett KA, Krueger JM. 2011 Biochemical regulation of sleep and sleep biomarkers. J Clin Sleep Med. 7:S38–S42. 22003330 10.5664/JCSM.1360 PMC3190410

[CIT0005] Faraut B, Boudjeltia KZ, Vanhamme L, Kerkhofs M. 2012 Immune, inflammatory and cardiovascular consequences of sleep restriction and recovery. Sleep Med Rev. 16:137–149. doi: 10.1016/j.smrv.2011.05.001 21835655

[CIT0006] Han JH, Kim DO, Yi JW, Park SW, Kang WJ, Choi YK, Kim S-H, Ko I-G, Jin J-J, Kim S-E, et al. 2014 Dexmedetomidine, α_2_-adrenoceptor agonist, does not induce apoptosis in the brachial plexus of rats. Anim Cells Syst. 18:407–415. doi: 10.1080/19768354.2014.983969

[CIT0007] Hu JF, Song XY, Chu SF, Chen J, Ji HJ, Chen XY, Yuan YH, Han N, Zhang JT, Chen NH. 2011 Inhibitory effect of ginsenoside Rg_1_ on lipopolysaccharide-induced microglial activation in mice. Brain Res. 374:8–14. doi: 10.1016/j.brainres.2010.11.069 21126513

[CIT0008] Hwang L, Choi IY, Kim SE, Ko IG, Shin MS, Kim CJ, Kim SH, Jin JJ, Chung JY, Yi JW. 2013 Dexmedetomidine ameliorates intracerebral hemorrhage-induced memory impairment by inhibiting apoptosis and enhancing brain-derived neurotrophic factor expression in the rat hippocampus. Int J Mol Med. 31:1047–1056. doi: 10.3892/ijmm.2013.1301 23503673

[CIT0009] Hwang L, Ko IG, Jin JJ, Kim SH, Kim CJ, Jeon JW, Han JH. 2018 *Scolopendra subspinipes mutilans* extract suppresses inflammatory and neuropathic pain *in vitro* and *in vivo*. Evid Based Complement Alternat Med. 17:5057372. 10.1155/2018/5057372 PMC6311788 30647762

[CIT0010] Jin JJ, Ko IG, Kim SE, Hwang L, Lee MG, Kim DY, Jung SY. 2017 Age-dependent differences of treadmill exercise on spatial learning ability between young- and adult-age rats. J Exerc Rehabil. 13:381–386. doi: 10.12965/jer.1735070.535 29114501 PMC5667613

[CIT0011] Kee N, Sivalingam S, Boonstra R, Wojtowicz JM. 2002 The utility of Ki-67 and BrdU as proliferative markers of adult neurogenesis. J Neurosci Methods. 115:97–105. doi: 10.1016/S0165-0270(02)00007-9 11897369

[CIT0012] Kempermann G, Kuhn HG, Gage FH. 1997 More hippocampal neurons in adult mice living in an enriched environment. Nature. 3:493–495. doi: 10.1038/386493a0 9087407

[CIT0013] Kim YM, Jin JJ, Lee SJ, Seo TB, Ji ES. 2018 Treadmill exercise with bone marrow stromal cells transplantation facilitates neuroprotective effect through BDNF-ERK1/2 pathway in spinal cord injury rats. J Exerc Rehabil. 14:335–340. doi: 10.12965/jer.1836264.132 30018915 PMC6028222

[CIT0014] Kim H, Lee SH, Kim SS, Yoo JH, Kim CJ. 2007 The influence of maternal treadmill running during pregnancy on short-term memory and hippocampal cell survival in rat pups. Int J Dev Neurosci. 25:243–249. doi: 10.1016/j.ijdevneu.2007.03.003 17434282

[CIT0015] Ko IG, Kim SE, Hwang L, Jin JJ, Kim CJ, Kim BK, Kim H. 2018 Late starting treadmill exercise improves spatial leaning ability through suppressing CREB/BDNF/TrkB signaling pathway following traumatic brain injury in rats. J Exerc Rehabil. 14:327–334. doi: 10.12965/jer.1836248.124 30018914 PMC6028205

[CIT0016] Lee JM, Ji ES, Kim TW, Kim CJ, Shin MS, Lim BV, Chung YR, Cho YS. 2018 Treadmill exercise improves memory function by inhibiting hippocampal apoptosis in pilocarpine-induced epileptic rats. J Exerc Rehabil. 14:713–723. doi: 10.12965/jer.36394.197 30443515 PMC6222143

[CIT0017] Li H, Yu F, Sun X, Xu L, Miu J, Xiao P. 2019 Dihydromyricetin ameliorates memory impairment induced by acute sleep deprivation. Eur J Pharmacol. 853:220–228. doi: 10.1016/j.ejphar.2019.03.014 30876981

[CIT0018] Lian H, Shim DJ, Gaddam SS, Rodriguez-Rivera J, Bitner BR, Pautler RG, Robertson CS, Zheng H. 2012 Iκbα deficiency in brain leads to elevated basal neuroinflammation and attenuated response following traumatic brain injury: implications for functional recovery. Mol Neurodegener. 7:47. doi: 10.1186/1750-1326-7-47 22992283 PMC3473257

[CIT0019] Moon EJ, Ko IG, Kim SE, Jin JJ, Hwang L, Kim CJ, An H, Lee BJ, Yi JW. 2018 Dexmedetomidine ameliorates sleep deprivation-induced depressive behaviors in mice. Int Neurourol J. 22:S139–S146. doi: 10.5213/inj.1836228.114 30396263 PMC6234724

[CIT0020] Murata Y, Oka A, Iseki A, Mori M, Ohe K, Mine K, Enjoji M. 2018 Prolonged sleep deprivation decreases cell proliferation and immature newborn neurons in both dorsal and ventral hippocampus of male rats. Neurosci Res. 131:45–51. doi: 10.1016/j.neures.2017.08.008 28865754

[CIT0021] Nelson LE, Lu J, Guo T, Saper CB, Franks NP, Maze M. 2003 The α^2^-adrenoceptor agonist dexmedetomidine converges on an endogenous sleep-promoting pathway to exert its sedative effects. Anesthesiology. 98:428–436. doi: 10.1097/00000542-200302000-00024 12552203

[CIT0022] Panzer O, Moitra V, Sladen RN. 2009 Pharmacology of sedative-analgesic agents: dexmedetomidine, remifentanil, ketamine, volatile anesthetics, and the role of peripheral mu antagonists. Crit Care Clin. 25:451–469. doi: 10.1016/j.ccc.2009.04.004 19576524

[CIT0023] Park JH, Ko IG, Kim SE, Jin JJ, Hwang L, Kim CJ, Yoon SH, Hong J, Chung JY, Lee DW. 2017 Dexmedetomidine oral mucosa patch for sedation suppresses apoptosis in hippocampus of normal rats. Int Neurourol J. 21:S39–S47. doi: 10.5213/inj.1734884.442 28446017 PMC5426424

[CIT0024] Sahu S, Kauser H, Ray K, Kishore K, Kumar S, Panjwani U. 2013 Caffeine and modafinil promote adult neuronal cell proliferation during 48 h of total sleep deprivation in rat dentate gyrus. Exp Neurol. 248:470–481. doi: 10.1016/j.expneurol.2013.07.021 23920241

[CIT0025] Sompol P, Liu X, Baba K, Paul KN, Tosini G, Iuvone PM, Ye K. 2011 N-acetylserotonin promotes hippocampal neuroprogenitor cell proliferation in sleep-deprived mice. Proc Natl Acad Sci U S A. 108:8844–8849. doi: 10.1073/pnas.1105114108 21555574 PMC3102377

[CIT0026] Sterniczuk R, Theou O, Rusak B, Rockwood K. 2013 Sleep disturbance is associated with incident dementia and mortality. Curr Alzheimer Res. 10:767–775. doi: 10.2174/15672050113109990134 23905991

[CIT0027] Tanabe K, Matsushima-Nishiwaki R, Kozawa O, Iida H. 2014 Dexmedetomidine suppresses interleukin-1β-induced interleukin-6 synthesis in rat glial cells. Int J Mol Med. 34:1032–1038. doi: 10.3892/ijmm.2014.1863 25069417

[CIT0028] Wadhwa M, Chauhan G, Roy K, Sahu S, Deep S, Jain V, Kishore K, Ray K, Thakur L, Panjwani U. 2018 Caffeine and modafinil ameliorate the neuroinflammation and anxious behavior in rats during sleep deprivation by inhibiting the microglia activation. Front Cell Neurosci. 12:49. doi: 10.3389/fncel.2018.00049 29599709 PMC5863523

[CIT0029] Wadhwa M, Prabhakar A, Ray K, Roy K, Kumari P, Jha PK, Kishore K, Kumar S, Panjwani U. 2017 Inhibiting the microglia activation improves the spatial memory and adult neurogenesis in rat hippocampus during 48 h of sleep deprivation. J Neuroinflammation. 14:222. doi: 10.1186/s12974-017-0998-z 29141671 PMC5688670

[CIT0030] Zhang X, Yan F, Feng J, Qian H, Cheng Z, Yang Q, Wu Y, Zhao Z, Li A, Xiao H. 2018 Dexmedetomidine inhibits inflammatory reaction in the hippocampus of septic rats by suppressing NF-κB pathway. PLoS One. 213:e0196897. doi: 10.1371/journal.pone.0196897 PMC5933780 29723264

[CIT0031] Zielinski MR, Krueger JM. 2011 Sleep and innate immunity. Front Biosci. 3:632–642. 10.2741/s176 PMC3645929 21196401

